# Investigation of Generator Rotor Dynamic Characteristics Under Unbalanced Electromagnetic Forces

**DOI:** 10.3390/s26113392

**Published:** 2026-05-27

**Authors:** Jiashun Dai, Hong Lu, Yukuo Guo, Hao Xue, Jiangnuo Mei, Qiong Wang

**Affiliations:** 1 School of Mechanical and Electronic Engineering, Wuhan University of Technology, Wuhan 430070, China; daijiashun@whut.edu.cn (J.D.); landzh@whut.edu.cn (H.L.); xueh100506@whut.edu.cn (H.X.); jiangnuomei@whut.edu.cn (J.M.); 2 China Merchants Heavy Industry (Jiangsu) Co., Ltd., Nantong 226100, China; guoyk_0807@163.com; 3 School of Mathematics and Statistics, Huazhong University of Science and Technology, Wuhan 430074, China; 4 Institute of Interdisciplinary Research for Mathematics and Applied Science, Huazhong University of Science and Technology, Wuhan 430074, China

**Keywords:** generator, air-gap eccentricity, unbalanced electromagnetic force, rotor dynamic response

## Abstract

With the increasing complexity of operating conditions and the trend toward structural compactness in generators, the unbalanced electromagnetic force induced by air-gap eccentricity has become a critical factor affecting rotor dynamic behavior and operational reliability. To address the strong coupling and modeling challenges among the electromagnetic field, mechanical force field, and lubrication flow field under eccentric conditions, this study proposes a multi-physics coupled modeling approach that integrates electromagnetic, structural, and fluid dynamic interactions. Based on the spatial pose characteristics of the rotor under eccentric conditions, a three-dimensional mathematical model of the air-gap length is established, and an analytical expression for the lubricating oil film thickness distribution is derived. This framework enables the coupled solution of unbalanced electromagnetic force, hydrodynamic oil film supporting force, and rotor dynamic response. A 60 kW-rated diesel generator was selected as the research object for both numerical simulations and experimental investigations. The numerical results indicate that when the load power increases from 0 kW to 60 kW, the displacement amplitude of the rotor in the y-direction increases by approximately 155%, demonstrating a significant enhancement of transverse vibration intensity under increasing unbalanced electromagnetic excitation. Comparison between experimental and numerical results shows good agreement in both variation trends and amplitude levels, with a maximum relative error of 4.07%, thereby validating the accuracy and reliability of the proposed electromagnetic–structural–fluid coupled model for predicting rotor dynamic response in generators.

## 1. Introduction

### 1.1. Research Purpose and Significance

As a key rotating component in power systems, the operational stability of a generator directly determines the efficiency and continuity of electrical energy supply. With the development of ship propulsion systems, rail transit, and large-scale industrial equipment toward higher power and higher rotational speeds, generator load levels have increased continuously, and internal structures have become increasingly compact. The resulting reduction in air-gap size significantly amplifies the influence of electromagnetic forces on the rotor dynamic response [1,2].

Under ideal operating conditions, the generator rotor and stator remain concentric, resulting in a uniform air-gap magnetic field distribution. In this case, radial electromagnetic forces balance each other and do not impose additional loads on the rotor [3]. However, in practical operation, the combined effects of manufacturing and assembly errors, initial shaft misalignment, bearing wear, thermal deformation, and electromagnetic parameter asymmetry often lead to pronounced non-uniformity in the air-gap magnetic field, thereby inducing unbalanced electromagnetic forces [4]. Engineering practice has demonstrated that unbalanced electromagnetic forces not only cause rotor orbit deviation, increased vibration amplitude, and deterioration of bearing loads, but also further induce a series of coupled faults, including oil film instability, rotor–stator rubbing, local overheating, and fatigue damage. In severe cases, these issues may even result in generator shutdowns and interruptions to power system operation [5,6].

Traditional rotor dynamics research has mainly focused on mechanical excitation or structural response analysis, while often neglecting the multi-physics coupling effects between electromagnetic forces and lubrication support characteristics. As a result, it is difficult to accurately describe the actual operating conditions of generator systems under complex working environments. With the rapid development of multi-physics coupling theory, numerical simulation methods, and online monitoring technologies, establishing a dynamic model that considers electromagnetic–structural–fluid coupling has become an important research direction for revealing the vibration mechanisms and performance degradation behavior of generators. Therefore, investigating the dynamic characteristics of generators under unbalanced electromagnetic forces can not only enrich the theoretical framework of multi-physics coupled rotor dynamics, but also provide a theoretical basis for generator condition monitoring and intelligent maintenance.

### 1.2. Related Works

Air-gap eccentricity refers to a typical mechanical operating condition in which the rotor axis is displaced relative to the stator axis. It is widely encountered in engineering practice and is difficult to eliminate completely [7]. Previous studies have shown that when the ratio of air-gap eccentricity to the rated air-gap length reaches approximately 10%, the air-gap magnetic field distribution becomes significantly distorted. The resulting unbalanced electromagnetic force increases markedly, strongly disturbing the lubrication oil film supporting state and consequently reducing generator operational stability [8,9].

Accurate calculation of unbalanced electromagnetic forces is fundamental to revealing the rotor dynamic response characteristics of generators under air-gap eccentricity conditions. In response to the magnetic field distortion induced by air-gap eccentricity and the associated electromagnetic force calculation, extensive studies have been conducted by researchers worldwide. Xu et al. [10] proposed an improved static air-gap eccentricity model to investigate the electromagnetic–mechanical characteristics of synchronous generators under eccentric conditions. Gou et al. [11] derived analytical expressions for air-gap permeance and magnetic flux density distributions under eccentricity, and analyzed the effects of tilt angle and axial position on the magnetic field distribution. Pourmoosa et al. [12] developed a modeling approach considering dynamic eccentricity and solved the electromechanical dynamic equations based on an air-gap permeance model, enabling effective prediction of key electromagnetic parameters under dynamic eccentricity conditions. Ma et al. [13] proposed a finite-element-based dynamic eccentricity modeling method to analyze harmonic magnetic fields and time-varying radial electromagnetic forces induced by eccentricity. Tao et al. [14] developed a dynamic eccentricity modeling approach based on finite-element analysis coupled with the Maxwell stress tensor method, and systematically investigated the evolution of harmonic magnetic fields, instantaneous electromagnetic forces, and unbalanced magnetic pull induced by dynamic eccentricity.

The oil film characteristics of journal bearings form the basis for describing their static and dynamic performance. Among these characteristics, accurate representation of the oil film thickness distribution serves as the geometric prerequisite for modeling, while efficient determination of the oil film pressure distribution constitutes the core of numerical analysis [15]. Deng et al. [16] investigated the effects of eccentricity ratio and length-to-diameter ratio through numerical analysis, and found that increasing eccentricity leads to a reduction in minimum oil film thickness and an increase in maximum oil film thickness, warning that excessive eccentricity may raise the risk of bearing contact due to overly thin oil films. Wu et al. [17] solved coupled governing equations incorporating thermal and rheological effects, demonstrating that temperature rise and elastic deformation jointly alter oil film distribution and load-carrying capacity. Zhang et al. [18] reported that misalignment not only induces axial variation in oil film thickness but also superimposes with eccentricity effects, causing a shift in the location of the minimum oil film thickness and a further reduction in its value. Shin et al. [19] developed a three-dimensional fluid–thermal–structural coupled finite element model to investigate the influence of journal misalignment on the Morton effect, with simulations indicating that misalignment angle amplitude has a significant impact on the extent of unstable regions. Dyk et al. [20] determined stability boundaries of journal center orbits using the frequency-domain response method, providing guidance for high-speed bearing design. Mallya et al. [21] employed a fully coupled time-domain approach to simulate transient bearing trajectories under eccentricity, impact disturbances, or sudden load variations, revealing the evolution of rotor orbits from circular and elliptical patterns to spiral and even chaotic trajectories. Song et al. [22] incorporated complex factors such as turbulence effects, oil film temperature rise, viscosity variation, elastic bearing deformation, dynamic eccentric displacement, and rotor misalignment, thereby enhancing the capability of nonlinear models to represent practical engineering operating conditions.

The unbalanced electromagnetic force generated during generator operation does not act on the supporting system in a one-way manner; instead, it is involved in a mutual coupling process mediated by rotor dynamic behavior. However, as summarized in Table 1, existing studies primarily focus either on electromagnetic field analysis or on lubrication characteristics of journal bearings, treating air-gap eccentricity as a prescribed input parameter. As a result, a closed-loop coupling relationship among air-gap eccentricity, unbalanced electromagnetic force, lubrication oil-film supporting force, and rotor dynamic response has not yet been fully established, which limits a comprehensive understanding of generator rotor dynamic behavior and operational stability.

### 1.3. Innovation and Contribution

To address the challenges of highly coupled electromagnetic characteristics, rotor dynamic responses, and lubrication fluid behavior under air-gap eccentricity conditions that are difficult to model in a unified framework, this study proposes a multi-physics coupling analysis method encompassing the generator’s electromagnetic field, structural field, and lubrication oil flow. Based on the developed “electromagnetic-structural-fluid” coupling model, the interactions among unbalanced electromagnetic forces, oil-film support characteristics, and rotor vibration responses are solved simultaneously.

The main innovations and technical contributions of this study are summarized as follows: (1) In Section 2, a mathematical model of the air-gap length was established to characterize the spatial posture of the rotor under eccentric conditions, enabling a parameterized representation of the air-gap geometry in three-dimensional space. Based on the ideal rigid-body assumption, an analytical expression for the oil-film thickness distribution under eccentric conditions was derived, providing a theoretical foundation for accurately modeling oil-film support characteristics under unbalanced electromagnetic forces. (2) In Section 3, a complete electromagnetic–structural–fluid coupling model of the generator was developed. The model systematically describes the magnetic field distortion caused by air-gap eccentricity, the time-varying characteristics of unbalanced electromagnetic forces, and the dynamic evolution of oil-film support. Furthermore, the model reveals the rotor dynamic response mechanism under multi-physics interactions. Through the experimental validation presented in Section 4, the predictive accuracy of the proposed model was quantitatively evaluated, demonstrating its significant technical value in rotor dynamic behavior analysis and generator operational stability assessment.

## 2. Analysis of Generator Electromagnetic Characteristics

### 2.1. Mathematical Model of Air-Gap Length

Under steady-state operating conditions, influenced by the dynamic characteristics of the supporting system, the displacement of the rotor axis relative to the stator axis typically exhibits periodic variations.

Experimental and practical studies have shown that when the ratio of air-gap eccentricity to the rated air-gap length reaches approximately 10%, the air-gap magnetic field distribution becomes significantly distorted, and the unbalanced electromagnetic forces increase markedly, resulting in a non-negligible impact on generator operational stability.

To gain deeper insight into the evolution of generator electromagnetic characteristics under eccentric operating conditions, it is necessary to construct a mathematical model of air-gap length that can accurately represent the relative spatial position of the rotor and stator. This allows parametric characterization of the air-gap length in three-dimensional space. By introducing key parameters such as eccentricity magnitude, angular displacement, and axial length, the spatial distribution of air-gap length can be systematically described, providing a solid foundation for subsequent calculations of electromagnetic parameters and analysis of unbalanced electromagnetic forces.

It should be noted that the air-gap length mathematical model established in this study is primarily applicable to conventional design parameters and normal steady-state operating conditions, in which the eccentricity remains within an engineering-acceptable range. Extreme air-gap eccentricity conditions, which may involve strongly nonlinear magnetic fields and breakdown of oil-film continuity, are not considered in the present work.

#### 2.1.1. Air-Gap Length Under Ideal Conditions

**Figure 1:**
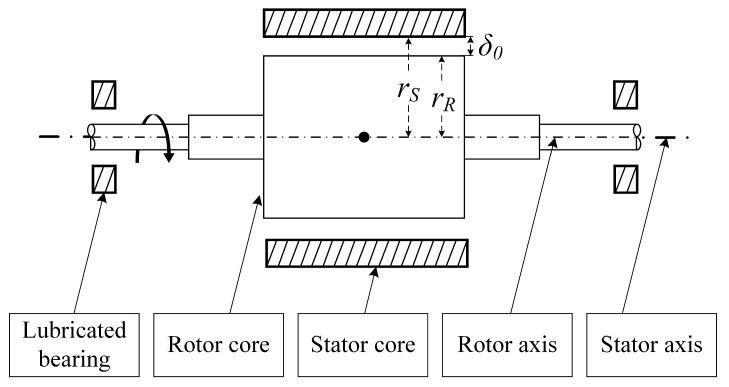
Air-gap distribution under ideal conditions.

Under ideal operating conditions, the rotor axis of the generator is perfectly aligned with the stator axis. The radial air-gap length exhibits axial symmetry in space and remains constant, so that the air gap can be regarded as a uniform annular clearance. Its geometric characteristics are determined jointly by the radii of the stator and rotor as well as manufacturing and assembly factors, as illustrated in Figure 1.

Under ideal operating conditions, the air-gap length is calculated as:

(1) $$\delta_{0}=r_{S}-r_{R}$$

where $r_{S}$ denotes the inner radius of the stator core, and $r_{R}$ represents the outer radius of the rotor core of the generator.

#### 2.1.2. Air-Gap Length Under Static Eccentricity

The air-gap eccentricity condition can be equivalently described as a coupled effect of translation and rotation of the rotor axis relative to the stator axis. Under translational displacement, the air-gap length exhibits circumferential compression and expansion. Under rotational misalignment, the air-gap length varies linearly along the axial direction, forming a wedge-shaped distribution. When these two effects coexist, the air-gap length is modulated through their coupling. In the circumferential region of minimum air gap, the angular misalignment further reduces the air gap at the axial near end while enlarging it at the far end. Conversely, in the region of maximum air gap, the opposite trend is observed.

**Figure 2:**
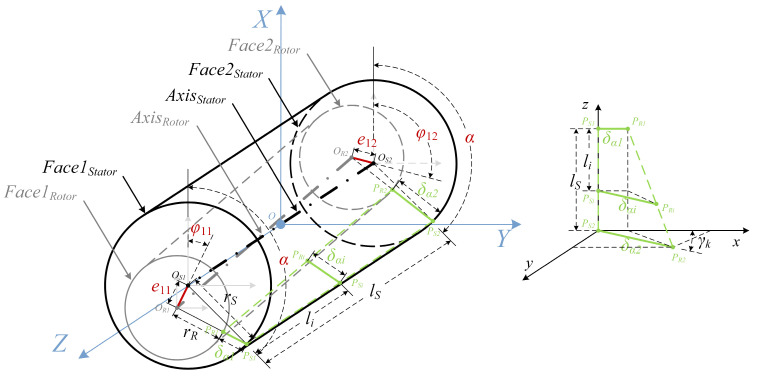
Air-gap distribution under static eccentricity.

After the occurrence of air-gap eccentricity, the originally ideal concentric annular air-gap structure is disrupted. The air-gap geometry evolves from an axisymmetric configuration into a three-dimensional structure with pronounced spatial asymmetry. Therefore, a mathematical model is established to parameterize the air-gap length at arbitrary spatial positions in three-dimensional space, as illustrated in Figure 2.

The distances between the rotor end-face centers $\left(O_{R1},O_{R2}\right)$ and the stator end-face centers $\left(O_{S1},O_{S2}\right)$ are defined as the eccentricity distances, denoted by $e_{11}$ and $e_{12}$, respectively. The angles between the lines connecting the stator and rotor end-face centers $\left(O_{S1}O_{R1},O_{S2}O_{R2}\right)$ and the X-axis are defined as the eccentricity angles, denoted by $\varphi_{11}$ and $\varphi_{12}$, respectively. The ratio of the eccentricity distance to the nominal air-gap length under ideal conditions is defined as the eccentricity ratio, denoted by $\chi_{11}$ and $\chi_{12}$, respectively.

(2) $$\left\{\begin{array}{l} \chi_{11}=\frac{e_{11}}{\delta_{0}}\\ \chi_{12}=\frac{e_{12}}{\delta_{0}} \end{array}\right.$$

Since $e_{11},e_{12}\ll r_{S}$, the effects of the small angles $∠O_{S1}P_{S1}O_{R1}$ and $∠O_{S2}P_{S2}O_{R2}$ can be neglected. Accordingly, the angles $∠O_{S1}O_{R1}P_{S1}$ and $∠O_{S2}O_{R2}P_{S2}$ are denoted by $\beta_{11}$ and $\beta_{12}$, respectively.

(3) $$\left\{\begin{array}{l} \beta_{11}=\alpha -\varphi_{11}\\ \beta_{12}=\alpha -\varphi_{12} \end{array}\right.$$

To describe the air-gap length at arbitrary positions on stator end faces ${Face1}_{\mathrm{Stator}}$ and ${Face2}_{\mathrm{Stator}}$ in a concise manner, a polar coordinate system is adopted. The centers of the stator inner circles, denoted by $O_{S1}$ and $O_{S2}$, are chosen as the poles, and the X-axis is taken as the polar axis.

Accordingly, the air-gap lengths corresponding to an arbitrary angular position α are denoted as $\delta_{\alpha 1}$ and $\delta_{\alpha 2}$, respectively. Their values can be obtained by applying the law of cosines to triangles $∆O_{S1}O_{R1}P_{S1}$ and $∆O_{S2}O_{R2}P_{S2}$.

(4) $$\left\{\begin{array}{l} \cos\beta_{11}=\frac{{e_{11}}^{2}+{\left(r_{R}+\delta_{\alpha 1}\right)}^{2}-{r_{S}}^{2}}{2e_{11}\left(r_{R}+\delta_{\alpha 1}\right)}\\ \cos\beta_{12}=\frac{{e_{12}}^{2}+{\left(r_{R}+\delta_{\alpha 2}\right)}^{2}-{r_{S}}^{2}}{2e_{12}\left(r_{R}+\delta_{\alpha 2}\right)} \end{array}\right.$$

The above expression is solved as follows.

(5) $$\left\{\begin{array}{l} \delta_{\alpha 1}=e_{11}\cos\beta_{11}-r_{R}\pm r_{S}\sqrt{1-{\left(\frac{e_{11}}{r_{S}}\right)}^{2}{\left(\sin\beta_{11}\right)}^{2}}\\ \delta_{\alpha 2}=e_{12}\cos\beta_{12}-r_{R}\pm r_{S}\sqrt{1-{\left(\frac{e_{12}}{r_{S}}\right)}^{2}{\left(\sin\beta_{12}\right)}^{2}} \end{array}\right.$$

Since $e_{11},e_{12}\ll r_{S}$, the terms $(e_{11}/r_{S}{)}^{2}{sin}^{2}\beta_{11}$ and $(e_{12}/r_{S}{)}^{2}{sin}^{2}\beta_{12}$ can be neglected. Taking the positive root, the air-gap length corresponding to an arbitrary angle α can be expressed as follows.

(6) $$\left\{\begin{array}{l} \delta_{\alpha 1}=\delta_{0}+e_{11}\cos\left(\alpha -\varphi_{11}\right)\\ \delta_{\alpha 2}=\delta_{0}+e_{12}\cos\left(\alpha -\varphi_{12}\right) \end{array}\right.$$

A three-dimensional coordinate system o-xyz is established to provide a unified reference frame. Point $P_{S2}$ is chosen as the origin, the direction of the vector $\vec{P_{S2}P_{S1}}$ is taken as the positive z-axis, and the direction of the vector $\vec{P_{S1}P_{R1}}$ is taken as the positive x-axis. The y-axis is then determined according to the right-hand rule.

Within this standardized coordinate system, the angle between vectors $\vec{P_{S1}P_{R1}}$ and $\vec{P_{S2}P_{R2}}$ is defined as $\gamma_{k}\left(k=1,\;2,\;3,\;4\right)$. Since the spatial relative positions of the two vectors may vary, the calculation of $\gamma_{k}$ can differ accordingly. To ensure both the rigor of the computational logic and the accuracy of the results, the analysis is carried out in a case-by-case manner based on the specific geometric relationships, under the assumption that $\phi_{11}\leq \phi_{12}$.

When $\left.\phi_{11}\in [0,\pi \right)$ and $\left.\phi_{12}\in [\phi_{11},\pi \right)$, $\gamma_{1}$ can be calculated as follows.

(7) $$\left\{\begin{array}{ll} \gamma_{1}= & \arccos\left(K_{11}\right)+\arccos\left(K_{12}\right)\\ & \alpha \in \left[\varphi_{11},\varphi_{12}\right)\cup \left[\varphi_{11}+\pi ,\varphi_{12}+\pi \right)\\ \gamma_{1}= & \left|\arccos\left(K_{11}\right)-\arccos\left(K_{12}\right)\right|\\ & \alpha \in \left[0,\varphi_{11}\right)\cup \left[\varphi_{12},\varphi_{11}+\pi \right)\cup \left[\varphi_{12}+\pi ,2\pi \right) \end{array}\right.$$

When $\left.\phi_{11}\in [0,\pi \right)$ and $\varphi_{12}\in \left[\pi ,\varphi_{11}+\pi \right)$, $\gamma_{2}$ can be calculated as follows.

(8) $$\left\{\begin{array}{ll} \gamma_{2}= & \arccos\left(K_{11}\right)+\arccos\left(K_{12}\right)\\ & \alpha \in \left[0,\varphi_{12}-\pi \right)\cup \left[\varphi_{11},\varphi_{12}\right)\cup \left[\varphi_{11}+\pi ,2\pi \right)\\ \gamma_{2}= & \left|\arccos\left(K_{11}\right)-\arccos\left(K_{12}\right)\right|\\ & \alpha \in \left[\varphi_{12}-\pi ,\varphi_{11}\right)\cup \left[\varphi_{12},\varphi_{11}+\pi \right) \end{array}\right.$$

When $\left.\phi_{11}\in [0,\pi \right)$ and $\varphi_{12}\in \left[\varphi_{11}+\pi ,2\pi \right)$, $\gamma_{3}$ can be calculated as follows.

(9) $$\left\{\begin{array}{ll} \gamma_{3}= & \arccos\left(K_{11}\right)+\arccos\left(K_{12}\right)\\ & \alpha \in \left[0,\varphi_{11}\right)\cup \left[\varphi_{12}-\pi ,\varphi_{11}+\pi \right)\cup \left[\varphi_{12},2\pi \right)\\ \gamma_{3}= & \left|\arccos\left(K_{11}\right)-\arccos\left(K_{12}\right)\right|\\ & \alpha \in \left[\varphi_{11},\varphi_{12}-\pi \right)\cup \left[\varphi_{11}+\pi ,\varphi_{12}\right) \end{array}\right.$$

When $\varphi_{11}\in \left[\pi ,2\pi \right)$ and $\varphi_{12}\in \left[\varphi_{11},2\pi \right)$, $\gamma_{4}$ can be calculated as follows.

(10) $$\left\{\begin{array}{ll} \gamma_{4}= & \arccos\left(K_{11}\right)+\arccos\left(K_{12}\right)\\ & \alpha \in \left[\varphi_{11}-\pi ,\varphi_{12}-\pi \right)\cup \left[\varphi_{11},\varphi_{12}\right)\\ \gamma_{4}= & \left|\arccos\left(K_{11}\right)-\arccos\left(K_{12}\right)\right|\\ & \alpha \in \left[0,\varphi_{11}-\pi \right)\cup \left[\varphi_{12}-\pi ,\varphi_{11}\right)\cup \left[\varphi_{12},2\pi \right) \end{array}\right.$$

In the above equation, the parameters $K_{11}$ and $K_{12}$ are defined as follows.

(11) $$\left\{\begin{array}{l} K_{11}=\frac{{r_{S}}^{2}+{\left(r_{R}+\delta_{\alpha 1}\right)}^{2}-{e_{11}}^{2}}{2r_{S}\left(r_{R}+\delta_{\alpha 1}\right)}\\ K_{12}=\frac{{r_{S}}^{2}+{\left(r_{R}+\delta_{\alpha 2}\right)}^{2}-{e_{12}}^{2}}{2r_{S}\left(r_{R}+\delta_{\alpha 2}\right)} \end{array}\right.$$

In the standardized coordinate system o-xyz, the coordinates of points $P_{R1}$ and $P_{R2}$ are given by $\left(\delta_{\alpha 1},0,l_{S}\right)$ and $\left(\delta_{\alpha 2}\cos\gamma_{k},\delta_{\alpha 2}\sin\gamma_{k},0\right)$, respectively. The two-point form equation is then expressed as follows.

(12) $$\frac{x-\delta_{\alpha 2}\cos\gamma_{k}}{\delta_{\alpha 1}-\delta_{\alpha 2}\cos\gamma_{k}}=\frac{y-\delta_{\alpha 2}\sin\gamma_{k}}{-\delta_{\alpha 2}\sin\gamma_{k}}=\frac{z}{l_{S}}$$

In the above equation, $l_{S}$ represents the axial length of the rotor core. The parametric equations for the coordinates of point $P_{Ri}$ are expressed as follows.

(13) $$\left\{\begin{array}{l} x\left(P_{Ri}\right)=\frac{l_{S}-l_{i}}{l_{S}}\left(\delta_{\alpha 1}-\delta_{\alpha 2}\cos\gamma_{k}\right)+\delta_{\alpha 2}\cos\gamma_{k}\\ y\left(P_{Ri}\right)=\frac{l_{S}-l_{i}}{l_{S}}\left(-\delta_{\alpha 2}\sin\gamma_{k}\right)+\delta_{\alpha 2}\sin\gamma_{k}\\ z\left(P_{Ri}\right)=l_{S}-l_{i} \end{array}\right.$$

For any cross-section parallel to ${Face1}_{\mathrm{Stator}}$ and located at a relative distance $l_{i}$, the air-gap length $\delta_{\alpha i}$ at an arbitrary angular position α within the section can be calculated as follows.

(14) $$\delta_{\alpha i}=\sqrt{{x\left(P_{Ri}\right)}^{2}+{y\left(P_{Ri}\right)}^{2}}$$

#### 2.1.3. Air-Gap Length Under Dynamic Eccentricity

In practical operation, the rotor axis of a generator rarely remains perfectly stationary. Its motion can be equivalently represented as a rotor axis with a certain eccentricity undergoing a periodic circular movement around the stator axis at a specific angular velocity, thereby forming a dynamic eccentricity state.

**Figure 3:**
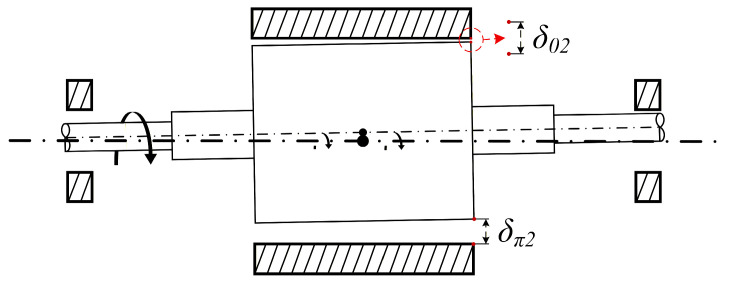
Air-gap distribution under dynamic eccentricity.

Under dynamic eccentricity, the instantaneous position of the rotor axis continuously evolves over time, and its trajectory in space forms a complex surface, as illustrated in Figure 3.

At a given time $t_{1}$, the eccentricities and orientation angles at the two end faces are denoted as $e_{11}$, $e_{12}$, $\phi_{11}$, and $\phi_{12}$, respectively, and the angular velocity of the rotor axis around the stator axis is denoted by $\omega_{R}$. After a time interval Δt, the eccentricity parameters at time $t_{2}$ are given by:

(15) $$\left\{\begin{array}{l} e_{21}\left(t_{2}\right)=e_{11}\\ e_{22}\left(t_{2}\right)=e_{12}\\ \varphi_{21}\left(t_{2}\right)=\varphi_{11}+\omega_{R}∆t\\ \varphi_{22}\left(t_{2}\right)=\varphi_{12}+\omega_{R}∆t \end{array}\right.$$

At time $t_{2}$, the air-gap lengths at any angular position α on the two stator end faces are given by:

(16) $$\left\{\begin{array}{l} \delta_{\alpha 1}\left(t_{2}\right)=\delta_{0}+e_{21}\left(t_{2}\right)\cos\left(\alpha -\varphi_{21}\left(t_{2}\right)\right)\\ \delta_{\alpha 2}\left(t_{2}\right)=\delta_{0}+e_{22}\left(t_{2}\right)\cos\left(\alpha -\varphi_{22}\left(t_{2}\right)\right) \end{array}\right.$$

In the standardized o-xyz coordinate system, the angle $\gamma_{k}(t_{2})$ between the vectors $\vec{P_{S1}P_{R1}}$ and $\vec{P_{S2}P_{R2}}$ can be calculated as follows.

(17) $$\gamma_{k}\left(t_{2}\right)=\&\left|\begin{array}{l} \arccos\left(\frac{{r_{S}}^{2}+{\left(r_{R}+\delta_{\alpha 1}\left(t_{2}\right)\right)}^{2}-{e_{21}\left(t_{2}\right)}^{2}}{2r_{S}\left(r_{R}+\delta_{\alpha 1}\left(t_{2}\right)\right)}\right)\\ \pm \arccos\left(\frac{{r_{S}}^{2}+{\left(r_{R}+\delta_{\alpha 2}\left(t_{2}\right)\right)}^{2}-{e_{22}\left(t_{2}\right)}^{2}}{2r_{S}\left(r_{R}+\delta_{\alpha 2}\left(t_{2}\right)\right)}\right) \end{array}\right|$$

To ensure continuity in the mathematical model and consistency with physical meaning, the choice of the positive or negative sign in equation (17) must be determined based on the specific values and relative relationships of the eccentricity parameters.

For any cross-section parallel to ${Face1}_{\mathrm{Stator}}$ and located at a relative distance $l_{i}$, the air-gap length at any angular position α at time $t_{2}$ is expressed as follows.

(18) $$\delta_{\alpha i}\left(t_{2}\right)=\sqrt{\begin{array}{l} {\left(\frac{l_{S}-l_{i}}{l_{S}}\left(\delta_{\alpha 1}\left(t_{2}\right)-\delta_{\alpha 2}\left(t_{2}\right)\cos\gamma_{k}\left(t_{2}\right)\right)+\delta_{\alpha 2}\left(t_{2}\right)\cos\gamma_{k}\left(t_{2}\right)\right)}^{2}\\ +{\left(\frac{l_{S}-l_{i}}{l_{S}}\left(-\delta_{\alpha 2}\left(t_{2}\right)\sin\gamma_{k}\left(t_{2}\right)\right)+\delta_{\alpha 2}\left(t_{2}\right)\sin\gamma_{k}\left(t_{2}\right)\right)}^{2} \end{array}}$$

### 2.2. Air-Gap Electromagnetic Parameters

#### 2.2.1. Air-Gap Magnetomotive Force

**Figure 4:**
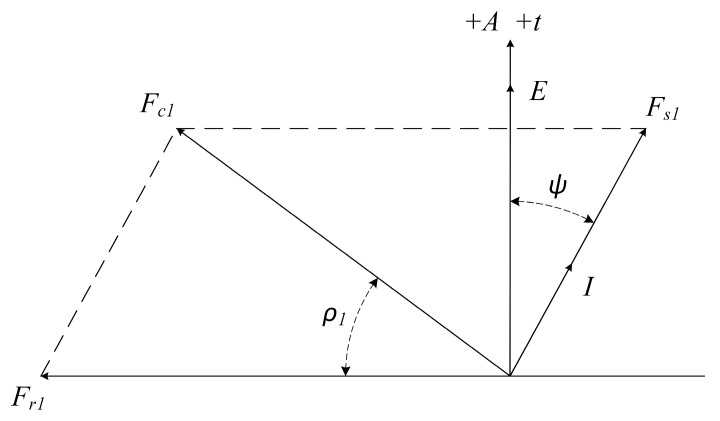
Vector superposition of air-gap MMF.

The air-gap magnetomotive force (MMF) is obtained by the vector superposition of the rotor winding MMF and the stator winding MMF, as illustrated in Figure 4.

The air-gap MMF mainly consists of n-th harmonic components (n=1, 3, 5, …). After vector superposition, the resulting expression of the air-gap MMF can be written as follows.

(19) $$\begin{array}{ll} f\left(\alpha ,t\right) & =\sum_{n=1,3,5\cdots }F_{rn}\cos\left(np\left(\omega_{r}t-\alpha \right)\right)+F_{sn}\cos\left(np\left(\omega_{r}t-\alpha \right)-\psi -\frac{\pi }{2}\right)\\ & =\sum_{n=1,3,5\cdots }F_{cn}\cos\left(n\left(\omega t-p\alpha -\rho_{n}\right)\right) \end{array}$$

In the above expression, $F_{rn}$ denotes the amplitude of the n-th harmonic component of the rotor winding magnetomotive force (MMF), $F_{sn}$ denotes the amplitude of the n-th harmonic component of the stator winding MMF, and $F_{cn}$ denotes the amplitude of the n-th harmonic component of the air-gap MMF. Here, ω represents the electrical angular velocity, p is the number of pole pairs, α is the circumferential angle, and $\rho_{n}$ denotes the phase angle between the n-th harmonic of the air-gap MMF and that of the rotor MMF.

#### 2.2.2. Air-Gap Permeability

The air-gap permeability per unit area is inversely proportional to the radial air-gap length. As a result, the air-gap permeability is relatively enhanced in regions with a minimum air-gap and reduced in regions with a maximum air-gap.

Under air-gap eccentricity conditions, the expression for the air-gap permeability per unit area is given as follows.

(20) $$\Lambda_{e}(t)=\frac{\mu_{0}}{\delta_{\alpha i}\left(t\right)}$$

where $\mu_{0}$ denotes the vacuum permeability, and $\delta_{\alpha i}(t)$ represents the radial air-gap length at a specific circumferential position at time t.

#### 2.2.3. Air-Gap Magnetic Density

Air-gap eccentricity mainly alters the radial air-gap length, thereby inducing spatiotemporal variations in the air-gap permeability, while its influence on the MMT generated by the windings is relatively small. Therefore, under air-gap eccentricity conditions, the spatiotemporal distribution of the air-gap magnetic density can be regarded as the product of the air-gap MMT under ideal conditions and the air-gap permeability under eccentric conditions.

The air-gap magnetic density under air-gap eccentricity can be expressed as follows.

(21) $$B_{e}\left(\alpha ,t\right)=f\left(\alpha ,t\right)\Lambda_{e}\left(t\right)$$

Under air-gap eccentricity, the magnetic density is relatively increased in regions near the minimum air-gap and reduced in regions near the maximum air-gap. Meanwhile, the frequency components remain similar to those under ideal conditions, consisting only of odd-order harmonics $\left(n=1,\;3,\;5,\;\ldots \right)$, with the fundamental harmonic being dominant.

### 2.3. Unbalanced Electromagnetic Force

When a material body is present in an electromagnetic field, its mechanical behavior manifests in the form of mechanical forces, referred to as electromagnetic force. Among the available approaches for analyzing electromagnetic force in electromagnetic fields, the Maxwell stress method provides the most realistic representation of the physical phenomena. By replacing the volumetric force with an equivalent magnetic traction, the electromagnetic force acting on the interface can be accurately evaluated.

Under air-gap eccentricity conditions, the radial electromagnetic force per unit area acting on the rotor surface is expressed as follows.

(22) $$q_{e}\left(\alpha ,t\right)=\&\frac{{B_{e}}^{2}\left(\alpha ,t\right)}{2\mu_{0}}=\frac{{\left[f\left(\alpha ,t\right)\Lambda_{e}(t)\right]}^{2}}{2\mu_{0}}$$

By integrating the radial electromagnetic force per unit area over the rotor circumference, the resulting unbalanced electromagnetic force acting on the rotor can be obtained as:

(23) $$\left\{\begin{array}{l} F_{em,X}(t)=l_{S}r_{R}\int_{0}^{2\pi }q_{e}\left(\alpha ,t\right)\cos\alpha \;d\alpha \\ F_{em,Y}(t)=l_{S}r_{R}\int_{0}^{2\pi }q_{e}\left(\alpha ,t\right)\sin\alpha \;d\alpha \end{array}\right.$$

## 3. Electromagnetic—Structural—Fluid Coupled Model

### 3.1. Multi-Physics Coupling Analysis

During actual operation, generators exhibit pronounced strong coupling among multiple physical fields. The presence of air-gap eccentricity breaks the spatial symmetry of the air-gap magnetic field, directly inducing and intensifying the unbalanced electromagnetic force. Acting as an additional load on the rotor system, this force continuously alters the dynamic response characteristics of the rotor.

As a critical fluid-support component of the rotor system, the load-carrying capacity of the lubricating oil film plays a decisive role in rotor dynamics. Under the excitation of unbalanced electromagnetic forces, the pressure and thickness distributions of the oil film evolve continuously, leading to dynamic variations in the rotor journal position. This, in turn, further modifies the air-gap eccentricity, amplifying both the magnitude and temporal complexity of the unbalanced electromagnetic force.

**Figure 5:**
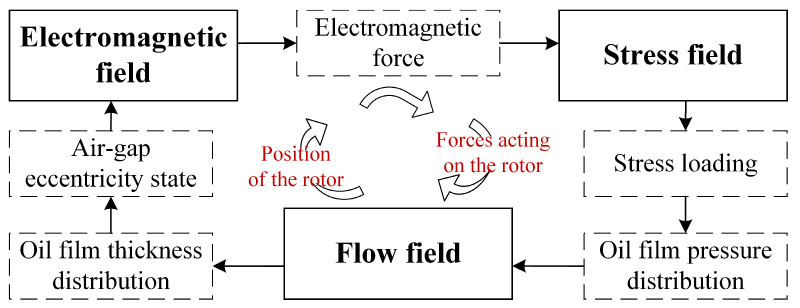
Multiphysics coupling relationships.

Figure 5 illustrates the cyclic evolution mechanism of the coupled interactions among the electromagnetic field, force field, and flow field in the generator. Under strong multi-physics coupling, these fields interact through unbalanced electromagnetic forces, oil film forces, and rotor motion responses, forming a feedback-regulated system that maintains a dynamic equilibrium under time-varying disturbance conditions.

A coupled electromagnetic–structural–fluid model for generators is established by integrating electromagnetic theory, rotor dynamics, and hydrodynamic lubrication theory. This model systematically reveals the coupling mechanisms and evolutionary characteristics among air-gap eccentricity, unbalanced electromagnetic force, oil-film supporting behavior, and rotor dynamic response, providing a unified theoretical framework for coupling mechanism investigation, operational stability analysis, and reliability assessment of generators.

### 3.2. Lubricating Oil-Film Supporting Characteristics Analysis

#### 3.2.1. Oil-Film Thickness Distribution Under Eccentric Conditions

The non-uniform distribution of the lubricating oil film thickness directly causes the rotor axis to deviate relative to the stator axis, thereby exerting a decisive influence on both the magnitude and temporal characteristics of the generator air-gap eccentricity. Meanwhile, the oil film thickness distribution, as a key parameter in the hydrodynamic lubrication mechanism, directly governs the formation and evolution of the oil film pressure distribution, which in turn determines the supporting capacity of the lubricating film.

Under typical design parameters and normal operating conditions, the generator shaft investigated in this study has a relatively short axial length and high structural stiffness. As a result, the elastic deformation induced by unbalanced electromagnetic forces and oil-film forces is generally much smaller than the air-gap size and can be regarded as a higher-order effect on the oil-film thickness distribution. Therefore, within the operating range considered in this study, neglecting rotor flexibility in the analytical expression of oil-film thickness does not alter the dominant characteristics of the oil-film pressure distribution or its load-carrying capability.

To reduce analytical complexity while ensuring physical plausibility and engineering applicability, the rotor is modeled as an ideal rigid body. That is, it is assumed that no bending or torsional deformation occurs during operation, and the rotor motion consists solely of rigid-body translation and rotation.

**Figure 6:**
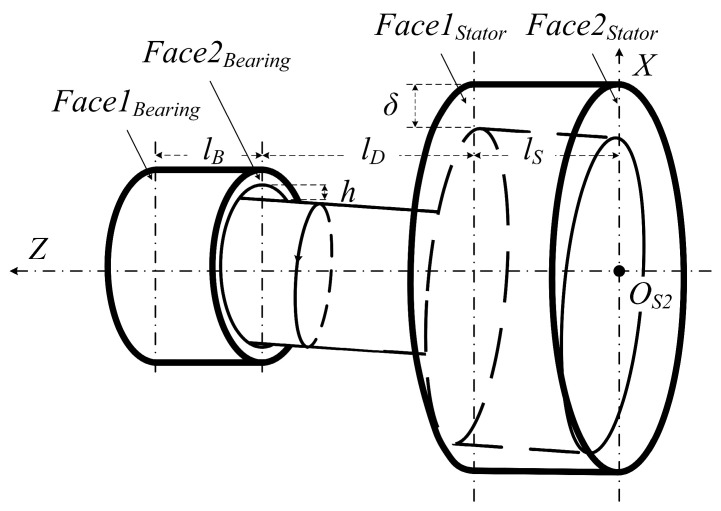
Lubricating oil-film thickness.

During actual operation, the rotor is subjected to multiple external loads, including gravity and unbalanced electromagnetic forces, which prevent the rotor axis from maintaining ideal coaxial alignment with the stator axis. Consequently, the rotor inevitably operates in a finite eccentric state. Based on the ideal rigid-body assumption and the air-gap length mathematical model, the lubricating oil film thickness parameters at time $t_{2}$ are derived, as shown in Figure 6.

Using the stator end-face ${\mathrm{Face}2}_{\mathrm{Stator}}$ center $O_{S2}$ as the origin, a three-dimensional coordinate system $O_{S2}\text{-}\mathrm{XYZ}$ is established. Neglecting the axial displacement effect of the rotor axis, the coordinates of points $O_{R1}$ and $O_{R2}$ are expressed as $\left(e_{11}\cos\left(\varphi_{11}+\pi \right),e_{11}\sin\left(\varphi_{11}+\pi \right),l_{S}\right)$, $\left(e_{12}\cos\left(\varphi_{12}+\pi \right),e_{12}\sin\left(\varphi_{12}+\pi \right),0\right)$, respectively. The two-point form equation is then expressed as follows.

(24) $$\frac{X-e_{12}\cos\left(\varphi_{12}+\pi \right)}{e_{11}\cos\left(\varphi_{11}+\pi \right)-e_{12}\cos\left(\varphi_{12}+\pi \right)}=\frac{Y-e_{12}\sin\left(\varphi_{12}+\pi \right)}{e_{11}\sin\left(\varphi_{11}+\pi \right)-e_{12}\sin\left(\varphi_{12}+\pi \right)}=\frac{Z}{l_{S}}$$

Let the rotor shaft centers within the bearing end-faces ${\mathrm{Face}1}_{\mathrm{Bearing}}$ and ${\mathrm{Face}2}_{\mathrm{Bearing}}$ be denoted as $O_{r1}$ and $O_{r2}$, respectively. The coordinates of both points satisfy Equation (24).

(25) $$\left\{\begin{array}{l} X\left(O_{r1}\right)=\frac{l_{S}+l_{D}+l_{B}}{l_{S}}\left(e_{11}\cos\left(\varphi_{11}+\pi \right)-e_{12}\cos\left(\varphi_{12}+\pi \right)\right)+e_{12}\cos\left(\varphi_{12}+\pi \right)\\ Y\left(O_{r1}\right)=\frac{l_{S}+l_{D}+l_{B}}{l_{S}}\left(e_{11}\sin\left(\varphi_{11}+\pi \right)-e_{12}\sin\left(\varphi_{12}+\pi \right)\right)+e_{12}\sin\left(\varphi_{12}+\pi \right)\\ Z\left(O_{r1}\right)=l_{S}+l_{D}+l_{B} \end{array}\right.$$

(26) $$\left\{\begin{array}{l} X\left(O_{r2}\right)=\frac{l_{S}+l_{D}}{l_{S}}\left(e_{11}\cos\left(\varphi_{11}+\pi \right)-e_{12}\cos\left(\varphi_{12}+\pi \right)\right)+e_{12}\cos\left(\varphi_{12}+\pi \right)\\ Y\left(O_{r2}\right)=\frac{l_{S}+l_{D}}{l_{S}}\left(e_{11}\sin\left(\varphi_{11}+\pi \right)-e_{12}\sin\left(\varphi_{12}+\pi \right)\right)+e_{12}\sin\left(\varphi_{12}+\pi \right)\\ Z\left(O_{r2}\right)=l_{S}+l_{D} \end{array}\right.$$

In the above equations, $l_{D}$ represents the axial distance between the stator end-face ${\mathrm{Face}1}_{\mathrm{Stator}}$ and the bearing end-face ${\mathrm{Face}2}_{\mathrm{Bearing}}$, while $l_{B}$ denotes the axial length of the bearing.

At time $t_{1}$, the eccentricity and angular displacement of the rotor shaft within the bearing end-faces ${\mathrm{Face}1}_{\mathrm{Bearing}}$ and ${\mathrm{Face}2}_{\mathrm{Bearing}}$ can be calculated as follows.

(27) $$\left\{\begin{array}{l} \overset{⃐}{e_{11}}\left(t_{1}\right)=\sqrt{{X\left(O_{r1}\right)}^{2}+{Y\left(O_{r1}\right)}^{2}}\\ \overset{⃐}{e_{12}}\left(t_{1}\right)=\sqrt{{X\left(O_{r2}\right)}^{2}+{Y\left(O_{r2}\right)}^{2}}\\ \overset{⃐}{\varphi_{11}}\left(t_{1}\right)=arccos\frac{X\left(O_{r1}\right)}{\sqrt{{X\left(O_{r1}\right)}^{2}+{Y\left(O_{r1}\right)}^{2}}}\;\left|Y\left(O_{r1}\right)\geq 0\right.\\ \overset{⃐}{\varphi_{11}}\left(t_{1}\right)=arccos\frac{X\left(O_{r1}\right)}{\sqrt{{X\left(O_{r1}\right)}^{2}+{Y\left(O_{r1}\right)}^{2}}}+\pi \;\left|Y\left(O_{r1}\right)<0\right.\\ \overset{⃐}{\varphi_{12}}\left(t_{1}\right)=arccos\frac{X\left(O_{r2}\right)}{\sqrt{{X\left(O_{r2}\right)}^{2}+{Y\left(O_{r2}\right)}^{2}}}\;\left|Y\left(O_{r2}\right)\geq 0\right.\\ \overset{⃐}{\varphi_{12}}\left(t_{1}\right)=arccos\frac{X\left(O_{r2}\right)}{\sqrt{{X\left(O_{r2}\right)}^{2}+{Y\left(O_{r2}\right)}^{2}}}+\pi \;\left|Y\left(O_{r2}\right)<0\right. \end{array}\right.$$

After a time interval Δt, at time $t_{2}$, the eccentricity parameters are given by:

(28) $$\left\{\begin{array}{l} \overset{⃐}{e_{21}}\left(t_{2}\right)=\overset{⃐}{e_{11}}\left(t_{1}\right)\\ \overset{⃐}{e_{22}}\left(t_{2}\right)=\overset{⃐}{e_{12}}\left(t_{1}\right)\\ \overset{⃐}{\varphi_{21}}\left(t_{2}\right)=\overset{⃐}{\varphi_{11}}\left(t_{1}\right)+\omega_{R}∆t\\ \overset{⃐}{\varphi_{22}}\left(t_{2}\right)=\overset{⃐}{\varphi_{12}}\left(t_{1}\right)+\omega_{R}∆t \end{array}\right.$$

Following the derivation approach of the air-gap length mathematical model, at time $t_{2}$, the hydrodynamic oil-film thickness at an arbitrary angle α within two bearing end-faces can be expressed as:

(29) $$\left\{\begin{array}{l} h_{\alpha 1}\left(t_{2}\right)=h_{0}+\overset{⃐}{e_{21}}\left(t_{2}\right)\cos\left(\alpha -\overset{⃐}{\varphi_{21}}\left(t_{2}\right)\right)\\ h_{\alpha 2}\left(t_{2}\right)=h_{0}+\overset{⃐}{e_{22}}\left(t_{2}\right)\cos\left(\alpha -\overset{⃐}{\varphi_{22}}\left(t_{2}\right)\right) \end{array}\right.$$

In the above equations, $h_{0}$ represents the radial clearance of the bearing, equal to the difference between the bearing radius $r_{b}$ and the journal radius $r_{r}$.

For any cross-section parallel to ${\mathrm{Face}1}_{\mathrm{Bearing}}$ at a distance $l_{j}$ from it, the oil-film thickness at an arbitrary angle α at time $t_{2}$, denoted as $h_{\alpha j}$, can be calculated as:

(30) $$h_{\alpha j}\left(t_{2}\right)=\sqrt{\begin{array}{l} {\left(\frac{l_{B}-l_{j}}{l_{B}}\left(h_{\alpha 1}\left(t_{2}\right)-h_{\alpha 2}\left(t_{2}\right)\cos\xi_{k}\left(t_{2}\right)\right)+h_{\alpha 2}\left(t_{2}\right)\cos\xi_{k}\left(t_{2}\right)\right)}^{2}\\ +{\left(\frac{l_{B}-l_{j}}{l_{B}}\left(-h_{\alpha 2}\left(t_{2}\right)\sin\xi_{k}\left(t_{2}\right)\right)+h_{\alpha 2}\left(t_{2}\right)\sin\xi_{k}\left(t_{2}\right)\right)}^{2} \end{array}}$$

In the above equations, the calculation of the angle parameter $\xi_{k}$ follows the same procedure as $\gamma_{k}$; detailed derivations are provided in Section 2.1.2 of this work.

#### 3.2.2. Oil-Film Pressure Distribution Under Eccentric Conditions

The core of hydrodynamic lubrication theory lies in characterizing the pressure distribution within the lubricating oil film. The fundamental governing equation is the partial differential equation describing the evolution of the oil-film pressure field. To reduce the equation’s complexity while maintaining physical fidelity, a series of simplifying assumptions is typically applied to the lubricating fluid. Based on these assumptions, the Navier–Stokes equations and the continuity equation can be systematically simplified and integrated, resulting in the Reynolds equation expressed in cylindrical coordinates:

(31) $$\frac{1}{{r_{b}}^{2}}\frac{\partial }{\partial \theta }\left(h^{3}\frac{\partial p}{\partial \theta }\right)+\frac{\partial }{\partial z}\left(h^{3}\frac{\partial p}{\partial z}\right)=6\mu \omega_{0}\frac{\partial h}{\partial \theta }+12\mu \frac{\partial h}{\partial t}$$

where h is the oil-film thickness, p is the oil-film pressure, μ is the dynamic viscosity of the lubricant, and $\omega_{0}$ is the angular velocity of the journal. The dimensionless form of the Reynolds equation is expressed as:

(32) $$\frac{\partial }{\partial \theta }\left(H^{3}\frac{\partial \bar{p}}{\partial \theta }\right)+{\left(\frac{r_{b}}{l_{B}}\right)}^{2}\frac{\partial }{\partial \bar{z}}\left(H^{3}\frac{\partial \bar{p}}{\partial \bar{z}}\right)=S_{f}\frac{\partial H}{\partial \theta }+2S_{f}\frac{\partial H}{\partial \tau }$$

In the above equations, $S_{f}$ represents the dimensionless speed coefficient.

**Figure 7:**
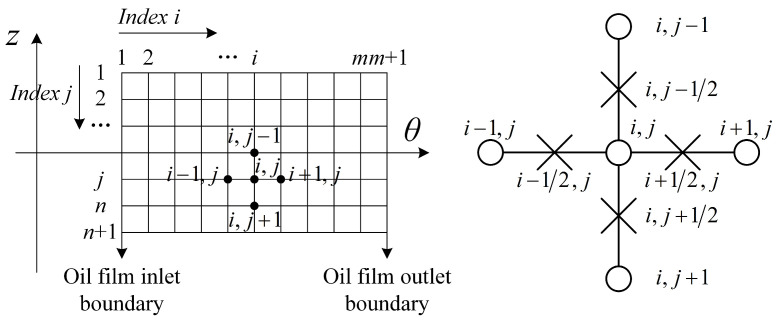
Mesh division and finite-difference representation of the lubricating oil-film.

The Reynolds equation is discretized using a half-step central difference scheme. The mesh division of the oil-film region and the finite-difference expressions are illustrated in Figure 7.

To simplify the calculation, the partial derivatives in the Reynolds equation are approximated by finite differences using the pressure values at the grid nodes. The pressure at each node is solved iteratively to obtain the pressure distribution across the oil film.

(33) $${\bar{p}}_{i,j}=\frac{A_{i,j}{\bar{p}}_{i+1,j}+B_{i,j}{\bar{p}}_{i-1,j}+C_{i,j}{\bar{p}}_{i,j+1}+D_{i,j}{\bar{p}}_{i,j-1}-F_{i,j}}{E_{i,j}}$$

Here, the coefficients $A_{i,j},B_{i,j},C_{i,j},D_{i,j},E_{i,j},F_{i,j}$ are defined as:

(34) $$\left\{\begin{array}{l} A_{i,j}=H_{i+1/2,j}^{3}\\ B_{i,j}=H_{i-1/2,j}^{3}\\ C_{i,j}={\left(\frac{r_{b}}{l_{B}}\cdot \frac{∆\theta }{∆\bar{z}}\right)}^{2}H_{i,j+1/2}^{3}\\ D_{i,j}={\left(\frac{r_{b}}{l_{B}}\cdot \frac{∆\theta }{∆\bar{z}}\right)}^{2}H_{i,j-1/2}^{3}\\ E_{i,j}=A_{i,j}+B_{i,j}+C_{i,j}+D_{i,j}\\ F_{i,j}=S_{f}\left(H_{i+1/2,j}-H_{i-1/2,j}\right)∆\theta +2S_{f}\left(\dot{X}\cos\theta +\dot{Y}\sin\theta \right){\left(∆\theta \right)}^{2} \end{array}\right.$$

The resulting system is solved using the successive over-relaxation method. Boundary nodes are assigned the known pressure values, and initial guesses are made for the internal nodes, denoted as ${\bar{p}}_{i,j}^{\left(1\right)}$ (i=2∼m, j=2∼n). Internal node pressures are then iteratively updated in sequence to improve the approximation. After k iterations, when the pressure distribution ${\bar{p}}_{i,j}^{\left(k\right)}$ is sufficiently accurate, the iteration is terminated.

To accelerate convergence, an over-relaxation factor β is introduced. The new value is computed as:

(35) $${\bar{p}}_{i,j}^{\left(k\right)}=\beta \left({\bar{p}}_{i,j}^{\left(k\right)}-{\bar{p}}_{i,j}^{\left(k-1\right)}\right)+{\bar{p}}_{i,j}^{\left(k-1\right)}$$

Typically, β is chosen in the range [1,2]. If it is too large, the solution may diverge. To determine whether the iterative solution has reached sufficient accuracy, a relative convergence criterion is used:

(36) $$\frac{\sum_{j=2}^{m}\sum_{i=2}^{n}\left|p_{i,j}^{\left(k\right)}-p_{i,j}^{\left(k-1\right)}\right|}{\sum_{j=2}^{m}\sum_{i=2}^{n}\left|p_{i,j}^{\left(k\right)}\right|}\leq \zeta$$

In the above equations, ζ is the allowed relative error, typically set to $10^{-3}$.

#### 3.2.3. Lubricating Oil-Film Supporting Force

When the rotor is subjected to an external load with known direction and magnitude, the lubricating oil film generates a corresponding supporting force in response to the applied load. Under steady operating conditions, and when the bearing operates within a stable lubrication regime, the oil film supporting force is, in an average sense, equal in magnitude and opposite in direction to the external load, thereby satisfying the force equilibrium condition of the rotor system.

**Figure 8:**
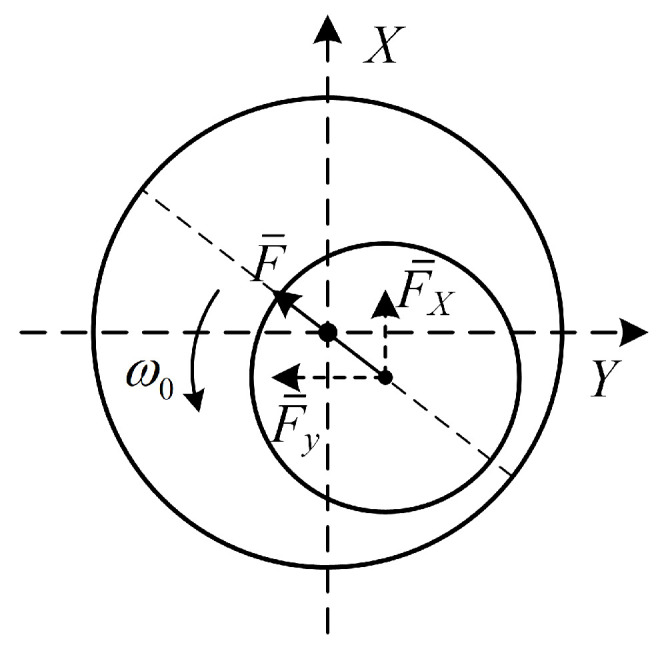
Vector decomposition of the oil-film supporting force in the Cartesian coordinate system.

As a key mechanical parameter characterizing the load-carrying capacity of sliding bearings, the oil film supporting force is illustrated in Figure 8. By integrating the oil film pressure along the horizontal and vertical directions, the supporting force can be expressed as a vector.

The dimensionless components of the supporting force along the X and Y directions are given by:

(37) $$\left\{\begin{array}{l} {\bar{F}}_{X}=\int_{0}^{1}\int_{0}^{2\pi }\bar{p}\cos\theta d\theta d\bar{z}\\ {\bar{F}}_{Y}=\int_{0}^{1}\int_{0}^{2\pi }\bar{p}\sin\theta d\theta d\bar{z} \end{array}\right.$$

The corresponding dimensional components of the oil-film supporting force along the horizontal and vertical directions are given by:

(38) $$\left\{\begin{array}{l} F_{h,X}=r_{b}l_{B}p_{0}{\bar{F}}_{X}\\ F_{h,Y}=r_{b}l_{B}p_{0}{\bar{F}}_{Y} \end{array}\right.$$

In the above equations, $p_{0}$ represents a characteristic pressure used for nondimensionalization, reflecting the typical magnitude of the oil film pressure variations.

### 3.3. Analysis of Rotor Dynamic Response Under Coupled Force Fields

Under the combined action of multiple external loads and the oil film supporting force, the rotor’s shaft center continuously evolves over time. By adjusting its instantaneous displacement, the rotor maintains overall force equilibrium, exhibiting a characteristic dynamic centering behavior.

**Figure 9:**
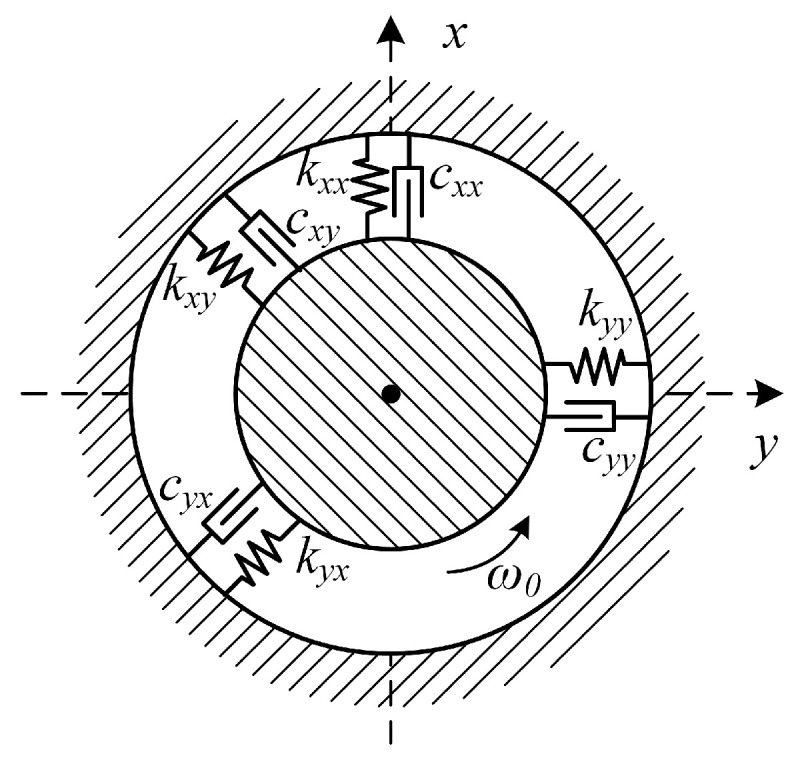
Dynamic model of the journal bearing–rotor system based on equivalent oil-film stiffness and damping.

To quantitatively describe the rotor’s dynamic response in the coupled force field, a sliding bearing dynamic model is constructed, using the oil film’s equivalent stiffness and equivalent damping coefficients as key parameters, as illustrated in Figure 9.

Taking the coordinate origin at the static equilibrium position, the oil film force can be considered as a function of the displacement and velocity near the equilibrium point. When the rotor center performs small-amplitude vibrations around the static equilibrium, the increment of the oil film force is expressed as:

(39) $$\left\{\begin{array}{l} ∆F_{h,X}=k_{xx}x+k_{xy}y+c_{xx}\dot{x}+c_{xy}\dot{y}\\ ∆F_{h,Y}=k_{yx}x+k_{yy}y+c_{yx}\dot{x}+c_{yy}\dot{y} \end{array}\right.$$

where $k_{xx},k_{xy},k_{yx},k_{yy}$ are the stiffness coefficients and $c_{xx},c_{xy},c_{yx},c_{yy}$ are the damping coefficients. These coefficients can be systematically determined using a finite difference method.

Considering that the rotor is subjected simultaneously to multiple external loads, such as unbalanced electromagnetic forces and gravity, the dynamic behavior of the rotor journal can be quantitatively described by the following equations of motion.

(40) $$\left\{\begin{array}{l} k_{xx}x+k_{xy}y+c_{xx}\dot{x}+c_{xy}\dot{y}+m\ddot{x}=F_{em,X}-mg\\ k_{yx}x+k_{yy}y+c_{yx}\dot{x}+c_{yy}\dot{y}+m\ddot{y}=F_{em,Y} \end{array}\right.$$

The electromagnetic force components at the rotor synchronous precession frequency $\omega_{0}$ can be decomposed into cosine and sine harmonic components $F_{C}(X)$, $F_{S}(X)$, $F_{C}(Y)$ and $F_{S}(Y)$, respectively.

(41) $$\left\{\begin{array}{l} F_{em,X}={\bar{F}}_{em,X}+{\tilde{F}}_{em,X}={\bar{F}}_{em,X}+F_{C}\left(X\right)\cos\left(\omega_{0}t\right)+F_{S}\left(X\right)\sin\left(\omega_{0}t\right)\\ F_{em,Y}={\bar{F}}_{em,Y}+{\tilde{F}}_{em,Y}={\bar{F}}_{em,Y}+F_{C}\left(Y\right)\cos\left(\omega_{0}t\right)+F_{S}\left(Y\right)\sin\left(\omega_{0}t\right) \end{array}\right.$$

Here, ${\bar{F}}_{em,X}$ and ${\bar{F}}_{em,Y}$ represent the DC components of the electromagnetic force, which together with gravity determine the steady-state equilibrium position of the rotor journal. ${\tilde{F}}_{em,X}$ and ${\tilde{F}}_{em,Y}$ are the periodic components.

The dynamic response of the rotor is synchronous with the external excitation frequency.

(42) $$\left\{\begin{array}{l} x\left(t\right)=\lambda_{1}\cos\left(\omega_{0}t\right)+\lambda_{2}\sin\left(\omega_{0}t\right)\\ y\left(t\right)=\lambda_{3}\cos\left(\omega_{0}t\right)+\lambda_{4}\sin\left(\omega_{0}t\right) \end{array}\right.$$

where $\lambda_{1},\;\lambda_{2},\;\lambda_{3},\;\lambda_{4}$ are displacement coefficients, calculated as:

(43) $$\left[\begin{array}{c} \lambda_{1}\\ \lambda_{2}\\ \lambda_{3}\\ \lambda_{4} \end{array}\right]={\left[\begin{array}{cc} \begin{array}{cc} k_{xx}-m{\omega_{0}}^{2} & c_{xx}\omega_{0}\\ -c_{xx}\omega_{0} & k_{xx}-m{\omega_{0}}^{2} \end{array} & \begin{array}{cc} k_{xy} & c_{xy}\omega_{0}\\ -c_{xy}\omega_{0} & k_{xy} \end{array}\\ \begin{array}{cc} k_{yx} & c_{yx}\omega_{0}\\ {-c}_{yx}\omega_{0} & k_{yx} \end{array} & \begin{array}{cc} k_{yy}-m{\omega_{0}}^{2} & c_{yy}\omega_{0}\\ -c_{yy}\omega_{0} & k_{yy}-m{\omega_{0}}^{2} \end{array} \end{array}\right]}^{-1}\left[\begin{array}{c} F_{C}\left(X\right)\\ F_{S}\left(X\right)\\ F_{C}\left(Y\right)\\ F_{S}\left(Y\right) \end{array}\right]$$

By iteratively updating the rotor journal’s dynamic response over discrete time steps, the time-evolving trajectory of the journal center can be obtained for the entire simulation period.

## 4. Numerical Simulation and Experimental Validation

### 4.1. Numerical Simulation

This section focuses on the dynamic response characteristics of the generator rotor under unbalanced electromagnetic forces. Matlab R2021b was employed to perform time-domain iterative numerical simulations of the dynamic model using a fixed time step. Through step-by-step coupled calculations of the unbalanced electromagnetic forces, oil-film forces, and rotor motion responses, the journal trajectories of the rotor under different load conditions were obtained. A diesel generator with a rated speed of 1500 rpm was selected as the study object, and the relevant parameters of the generator and lubricating bearings are summarized in Table 2.

The load power ranges from 0 to 60 kW and is divided into four equally spaced representative operating conditions: 0 kW, 20 kW, 40 kW, and 60 kW. For these four representative operating conditions, time-domain iterative simulations were carried out using a fixed time step of 0.00001 s. To verify the rationality of the selected time step, a numerical convergence analysis was further performed. By comparing the rotor displacement responses under different time-step conditions, the results indicate that, with the adopted time step, both the rotor displacement amplitudes and shaft center trajectories tend to converge to stable values, thereby achieving a good balance between computational efficiency and numerical accuracy.

A periodic convergence analysis was performed on the rotor displacement responses in the two orthogonal directions, x and y. The results indicate that, for two consecutive rotational periods, the displacement amplitudes in both directions satisfied the prescribed convergence criterion; specifically, the relative difference in amplitude between adjacent periods, defined as the ratio of the amplitude difference to that of the preceding period, was less than 1%.

**Figure 10:**
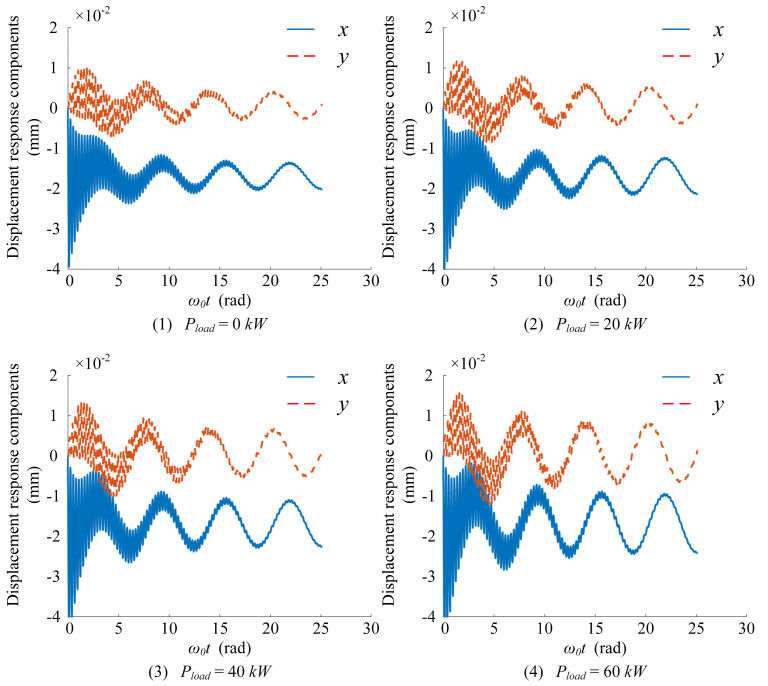
Displacement response components.

**Figure 11:**
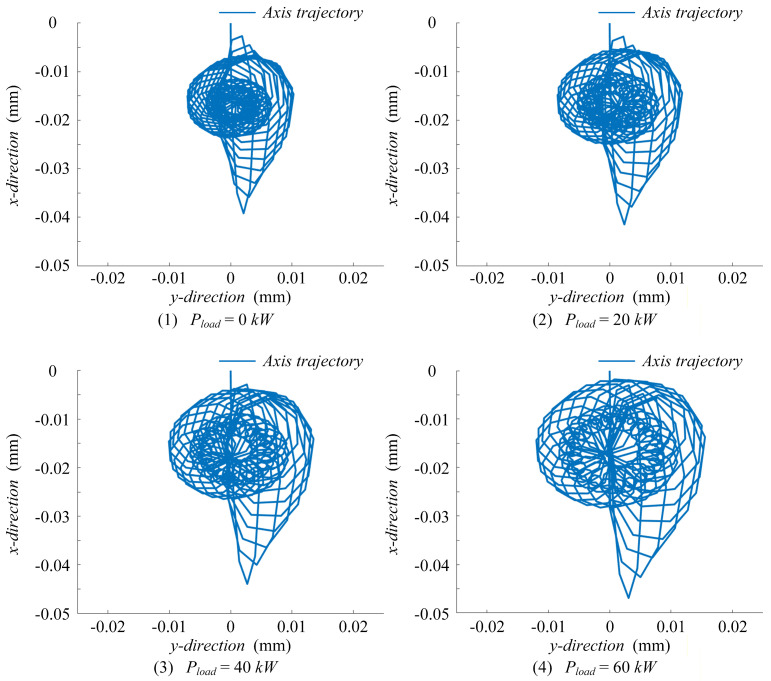
Axis trajectory.

Accordingly, the system can be considered to have reached a stable periodic response under all investigated operating conditions. The corresponding displacement response curves are presented in Figure 10. Based on the converged x- and y-direction displacement components, the shaft center trajectories were reconstructed, as shown in Figure 11.

When the load power increases from 0 kW to 60 kW, the rotor displacement responses in the two orthogonal directions, x and y, exhibit pronounced periodic fluctuations, with amplitudes increasing monotonically as the load power rises. Specifically, the displacement amplitude in the y-direction increases from $0.259\times 10^{-2}$ mm to $0.661\times 10^{-2}$ mm, while that in the x-direction increases from $2.002\times 10^{-2}$ mm to $2.386\times 10^{-2}$ mm. Moreover, the displacement amplitude in the x-direction is significantly larger than that in the y-direction, indicating that the lubricating oil film provides a dominant supporting force component along the x-direction.

From the perspective of periodic behavior, as the load power increases, the fundamental period of the displacement response remains consistent with the rotor rotational frequency, whereas the vibration amplitude and the major axis of the orbital trajectory gradually increase, indicating a progressively intensified radial vibration response. This trend suggests that an increase in load power enhances the air-gap magnetic field strength between the stator and rotor, thereby increasing the magnitude of the unbalanced electromagnetic force. Under air-gap eccentricity conditions, this radial unbalanced electromagnetic force acts as a periodic excitation source, continuously acting on the rotor system and amplifying its lateral dynamic response.

To maintain force equilibrium, the lubricating oil film adjusts its pressure distribution to enhance its load-carrying capacity, which is reflected by a gradual reduction in the minimum oil film thickness and a corresponding increase in peak oil film pressure. However, within the investigated load range, the bearing remains in a stable hydrodynamic lubrication regime. Under full-load conditions, the journal eccentricity ratio reaches 47.72%, which falls within the acceptable range predicted by classical hydrodynamic lubrication theory, indicating that stable oil film support is maintained without breakdown or instability. Therefore, although increasing load intensifies the amplitude of the periodic vibration, the system continues to operate in a stable periodic state, and the numerical simulation results are in good agreement with established theoretical assumptions.

### 4.2. Experimental Validation

To verify the engineering applicability of the proposed “electromagnetic-structural-fluid” coupled model, dynamic response experiments of the generator rotor were conducted using a diesel generator test bench equipped with a data acquisition and analysis system. Rotor radial displacement data under four typical load conditions were collected, processed, and analyzed. The experimental results were then compared with numerical simulation results to systematically evaluate the influence of load variation on the rotor’s dynamic response characteristics.

**Figure 12:**
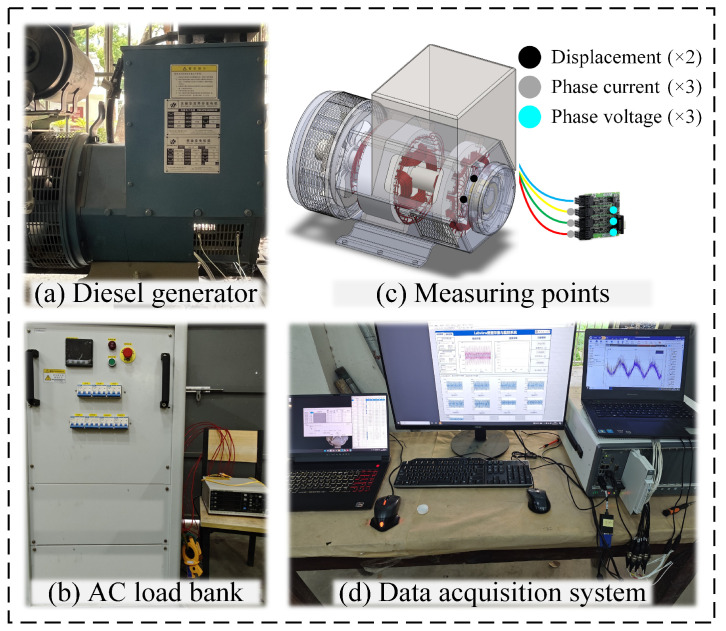
Diesel generator and data acquisition system.

Figure 12 shows the diesel generator test bench and the data acquisition and analysis system. To enable coordinated collection and synchronized processing of multiple parameters, a high-precision integrated data acquisition and analysis system was developed, incorporating multi-channel current, voltage, and displacement measurement modules to ensure the completeness and accuracy of the experimental data.

The current and voltage measurement module consists of a PW-3337 power analyzer and 9661 clamp-type current sensors, with a measurement error of ±0.15% of the reading. The measured signals include the three-phase AC current and AC voltage output from the diesel generator. The phase currents were measured by clamping the U, V, and W phase cables, while the phase voltages were measured using a three-phase four-wire connection method. To reduce the interference of electromagnetic signals on the data acquisition system, shielding and grounding treatments were applied to the sensor wiring during the experiments.

The displacement measurement module comprises a PXIe-4499 data acquisition device and YSV3308 eddy current displacement sensors, with a measurement error of ±0.05% of full scale. The displacement sensors were arranged near the bearing end in both the vertical and horizontal directions. By combining the displacement signals from these two orthogonal directions, the rotor shaft center trajectory can be effectively reconstructed.

During the data acquisition process, all relevant response variables were synchronously recorded after the diesel generator reached steady-state operating conditions. The data acquisition frequency was set to 10 kHz, and the data collected continuously over a 30 s interval were subjected to time-averaging and statistical analysis. The corresponding results are summarized in Table 3.

To establish a reference mechanical response without electromagnetic excitation and to characterize the inherent dynamic properties of the bearing–rotor system, Test Group 1 was designed under no-excitation, no-load conditions, whereas Test Groups 2–4 were performed under loaded operating conditions. As the load power increases from 0 kW to 60 kW, the displacement amplitude along the y-direction rises from 2.68 μm to 6.87 μm, while that along the x-direction increases from 20.58 μm to 24.83 μm. The displacement amplitudes of the rotor shaft center in both directions grow with increasing load, indicating that load variations lead to changes in the unbalanced electromagnetic forces, which in turn have a significant impact on the dynamic response of the generator rotor.

On this basis, the experimental results were compared with the numerical simulation results, as summarized in Table 4. Under the four typical load conditions, the experimental data show good agreement with the simulation results in both trend and magnitude. Although the relative error slightly increases with load, it remains below 5% in all cases, confirming the accuracy of the proposed coupled model in predicting the rotor’s dynamic response under varying load conditions.

## 5. Conclusions and Future Work

During generator operation, the unbalanced electromagnetic forces induced by dynamic air-gap eccentricity vary periodically, continuously perturbing the stability of the rotor’s oil-film support. To address this issue, this study focuses on the multi-physics coupling relationships within the generator and combines theoretical modeling, numerical simulation, and experimental validation to systematically investigate the rotor’s dynamic response under eccentric conditions, considering unbalanced electromagnetic forces, oil-film support forces, and their coupled interactions. The main conclusions are as follows:

(1) The developed mathematical model of the air-gap length under eccentric conditions enables a three-dimensional parameterized representation of the rotor spatial posture and air-gap geometry. Based on the ideal rigid-body assumption, an analytical expression for the oil-film thickness distribution under eccentric conditions was derived. On this basis, an electromagnetic–structural–fluid multi-physics coupling model was established, revealing the rotor dynamic response mechanism under multi-physics interactions.
(2) As the load power increases, the amplitude of unbalanced electromagnetic forces gradually rises, leading to a significant increase in the rotor’s radial displacement response. The degree of dynamic eccentricity also increases, further amplifying the variation in unbalanced electromagnetic force, indicating a pronounced coupling effect between load changes and dynamic eccentricity.
(3) Under stable support conditions, the oil film adjusts its pressure distribution to enhance load-bearing capacity, as reflected by a decrease in the minimum film thickness and an increase in oil-film pressure. This adaptive behavior effectively maintains the system’s mechanical balance and operational stability.
(4) The developed electromagnetic–structural–fluid multi-physics coupled model accurately characterizes the rotor’s dynamic response under load variations and unbalanced electromagnetic forces, providing a reliable theoretical basis for predicting rotor dynamics and analyzing operational stability in generators.

Future research can build upon the existing theoretical framework to extend the analysis to non-steady-state operating conditions, such as startup, shutdown, sudden load changes, and overload. This would enable a systematic investigation of the transient evolution of unbalanced electromagnetic forces and their impact on rotor dynamic behavior. In addition, incorporating nonlinear factors such as oil film temperature rise, turbulence effects, and bearing elastic deformation can enable the development of a higher-fidelity multi-physics coupled model, enhancing its applicability to real-world engineering conditions.

## Figures and Tables

**Table 1:** Comparative analysis of existing studies.

Reference	Physical Fields Considered	Air-Gap Eccentricity Modeling	Lubrication Oil-Film Modeling	Rotor Dynamic Modeling	Main Limitations
Xu et al. [10]	Electromagnetic, Mechanical	Static eccentricity	Not considered	Simplified dynamic model	Dynamic air-gap variation and lubrication effects not included
Gou et al. [11]	Electromagnetic	Static eccentricity	Not considered	Not considered	Lacks coupling with rotor dynamics and bearing system
Pourmoosa et al. [12]	Electromagnetic, Mechanical	Dynamic eccentricity	Not considered	Included	Bearing lubrication effects neglected
Ma et al. [13]	Electromagnetic	Dynamic eccentricity	Not considered	Not considered	Electromagnetic forces not coupled with rotor-bearing dynamics
Tao et al. [14]	Electromagnetic	Dynamic eccentricity	Not considered	Not considered	No feedback of electromagnetic forces to rotor response
Deng et al. [16]	Fluid lubrication	Prescribed eccentricity ratio	Included	Not considered	Eccentricity treated as an input parameter, no electromagnetic
Wu et al. [17]	Fluid, Thermal, Structural	Prescribed eccentricity	Included	Not considered	Electromagnetic excitation source not considered
Zhang et al. [18]	Fluid lubrication	Eccentricity, misalignment	Included	Not considered	Lack of electromagnetic–mechanical coupling
Shin et al. [19]	Fluid, Thermal, Structural	Prescribed misalignment	Included	Simplified	No air-gap electromagnetic constraints
Dyk et al. [20]	Fluid, Mechanical	Prescribed eccentricity	Included	Included	Eccentricity not linked to electromagnetic forces
Mallya et al. [21]	Fluid, Mechanical	Prescribed eccentricity	Included	Included	External excitation assumed, electromagnetic effects ignored
Song et al. [22]	Fluid, Thermal, Structural	Dynamic eccentricity	Included	Included	Air-gap electromagnetic field not explicitly modeled

**Table 2:** Parameters of the generator and lubricating bearings.

Generator Parameter	Value/Units	lubricating Bearings Parameter	Value/Units
Rated power	60 kW	Lubricating oil viscosity	0.015 Pa·s
Rated voltage	400 V	Lubricating oil density	$876.5\;kg/m^{3}$
Stator axial length	240 mm	Bearing axial length	60 mm
Stator inner radius	138 mm	Bearing inner radius	40 mm
Air-gap length	2 mm	Radial clearance	0.05 mm

**Table 3:** Experimental results under various operating conditions.

Test Group	Phase Current (A)			Phase Voltage (V)			Displacement Amplitude (x) (μm)	Displacement Amplitude (y) $\left(\mu m\right)$
	U	V	W	U	V	W		
1	0	0	0	1.07	1.18	0.98	20.58	2.68
2	30.52	30.35	30.19	220.18	219.75	219.71	21.95	3.94
3	60.77	60.66	60.53	220.77	220.39	220.21	23.36	5.24
4	90.90	90.77	90.62	220.98	220.60	220.77	24.83	6.87

**Table 4:** Comparison between numerical simulation and experimental results.

Load Power (kW)	Simulation Results (μm)		Experimental Results (μm)		Relative Error (%)	
	*x*-Axis	*y*-Axis	*x*-Axis	*y*-Axis	*x*-Axis	*y*-Axis
0	20.02	2.59	20.58	2.68	2.80%	3.47%
20.02	21.24	3.80	21.95	3.94	3.34%	3.68%
40.11	22.52	5.05	23.36	5.24	3.73%	3.76%
60.12	23.86	6.61	24.83	6.87	4.07%	3.93%

## Data Availability

The data presented in this study are available on request from the corresponding author.
